# MiR-181a: a potential biomarker of acute muscle wasting following elective high-risk cardiothoracic surgery

**DOI:** 10.1186/s13054-015-0853-5

**Published:** 2015-04-07

**Authors:** Susannah AA Bloch, Anna VJ Donaldson, Amy Lewis, Winston AS Banya, Michael I Polkey, Mark JD Griffiths, Paul R Kemp

**Affiliations:** Molecular Medicine, National Heart and Lung Institute, Imperial College, SW7 2AZ London, UK; National Institute for Health Research (NIHR) Respiratory Biomedical Research Unit at the Royal Brompton & Harefield NHS Foundation Trust and Imperial College, SW3 6NP London, UK; Leukocyte Biology, National Heart and Lung Institute, Imperial College London, London, UK

## Abstract

**Introduction:**

Acute muscle wasting in the critically ill is common and associated with significant morbidity and mortality. Although some aetiological factors are recognised and muscle wasting can be detected early with ultrasound, it not possible currently to predict in advance of muscle loss those who will develop muscle wasting. The ability to stratify the risk of muscle wasting associated with critical illness prior to it becoming clinically apparent would provide the opportunity to predict prognosis more accurately and to intervene at an early stage. MicroRNAs are small non-coding RNAs that modulate post-transcriptional regulation of translation, some are tissue specific and can be detected and quantified in plasma. We hypothesised that certain plasma microRNAs could be biomarkers of ICU acquired muscle weakness.

**Methods:**

Plasma levels of selected microRNAs were measured in pre- and post-operative samples from a previously reported prospective observational study of 42 patients undergoing elective high-risk cardiothoracic surgery, 55% of whom developed muscle wasting.

**Results:**

The rise in miR-181a was significantly higher on the second post-operative day in those who developed muscle wasting at 1 week compared to those who did not (p = 0.03). A rise in miR-181a of greater than 1.7 times baseline had 91% specificity and 56% sensitivity for subsequent muscle wasting. Other microRNAs did not show significant differences between the groups.

**Conclusion:**

Plasma miR-181a deserves further investigation as a potential biomarker of muscle wasting. Additionally, since mir-181a is involved in both regulation of inflammation and muscle regeneration and differentiation; our observation therefore also suggests directions for future research.

## Introduction

Acute muscle wasting following critical illness is a common clinical problem [1], which is associated with significant morbidity and mortality [2]. Damage to muscle function and structure starts in the acute phase of the clinical illness [3,4]. The muscle wasting and consequent loss of strength that results from critical illness varies both in severity and duration. Overall in critically ill patients who are intubated for a week or more, 25% have significant muscle weakness at awakening [5], but in the worst cases patients may become severely disabled requiring long-term respiratory weaning and developing a long-lasting neuromuscular deficit [6].

Muscle wasting and weakness although related are not directly comparable, however, the ability to predict the development of muscle wasting prior to its development, or before the patient can be assessed for weakness would enable clinicians to predict prognosis, direct early rehabilitation and guide management, which may in the future include anabolic drugs [7]. Although early muscle wasting can be detected with ultrasound [4] this may be unavailable or technically challenging and therefore a circulating biomarker would have significant advantages, especially if changes preceded loss of muscle cross-sectional area. Lastly a predictive biomarker of acute muscle wasting would facilitate selection of high-risk patients and thus, allow enrichment of stratified medicine trials.

MicroRNAs are small non-coding RNAs that modify translation. Many are tissue-specific and can be quantified reliably and reproducibly in blood [8-10]. The role of microRNAs in the pathogenesis of disease is a fast expanding area of research. However, their potential use as biomarkers is also increasingly recognised, as they circulate in blood, are often tissue-specific and are resistant to breakdown as they are believed to circulate in protective exosomes [10,11], for example, as prognostic and diagnostic markers in acute coronary syndromes [9] and in diagnosis of solid and haematological cancers [11].

Our group has previously shown that blood microRNA profile reflects muscle phenotype and muscle atrophy in chronic obstructive pulmonary disease (COPD) [12,13]. Therefore, we hypothesised that muscle-specific microRNAs, which are known to be involved in muscle homeostasis and metabolism (1,133,206,499 [14]) and have been found to be associated with muscle wasting by our group [12,13], as well as those involved in the regulation of muscle regeneration and inflammation (30b, 181a - believed to be the major member of the 181 family functional in muscle [14,15]) might prove to be useful biomarkers of early development of acute muscle wasting in the critically ill patient. Last, the role for these microRNAs in particular as a biomarker was suggested by our recent observation that muscle expression of these microRNAs is reduced in patients with established ICU-acquired muscle wasting [16].

## Material and methods

### Patient recruitment and study design

Blood from a previously reported prospective observational study was used [17]. Briefly patients undergoing a high-risk elective procedure requiring post-operative admission to adult critical care were recruited. Patients involved in the study gave informed written consent to take part. The study was ethically approved by the local research ethics committee approval under the National Institute for Health Research (NIHR) Respiratory Biomedical Research Unit (BRU) at the Royal Brompton and Harefield National Health Service (NHS) trust (10/H0504/9). High-risk patients were defined by the surgical team and EuroSCORE assessment [18]. Malignancy, pre-existing muscle or neuromuscular disease, or contraindication to serial blood sampling excluded patients from the study. Measurement of the cross-sectional area of the right rectus femoris muscle by ultrasound (US RF_csa_) (see below) was used to assess quadriceps muscle mass pre-operatively, on the day before surgery. This was repeated at day 7 of admission or on discharge from hospital if earlier than day 7. Bloods were taken pre-operatively for analysis (baseline), on the 1st (D1) and 2nd (D2) post-operative days and at day 7 or at discharge from hospital if earlier (D7). D7 and discharge earlier than day 7 were considered and analysed as the same time point: this only applied to three patients in the non muscle-wasting group and one in the muscle-wasting group. Following centrifuge separation of plasma (1,500 g for 10 minutes) samples were aliquoted and stored at −80°C within 2 hours of collection.

### Definition of quadriceps wasting by measurement of cross-sectional area of the rectus femoris by ultrasound

B-mode ultrasound (US) imaging with a 10-MHz 12 L-RS probe was used (Logiq E, GE Healthcare, Buckinghamshire, UK) to measure the US RF_csa_ as previously described [17,19,20]. Three separate images were obtained and the RF_csa_ estimated using Image-J software (National Institutes of Health, USA). Either the point 2/3 or 4/5 of the distance from the anterior superior iliac spine to the superior border of the patella (both points have been validated by our group) was used to measure the US RF_csa,_ and the same point measured for repeated measures. The mean of these three measurements was used. Images were excluded (n = 3) if the edges of the RF muscle could not be defined well enough to calculate the RF_csa,_ for example if the patient was very oedematous. Patients were classified as those who developed muscle wasting if they developed more than 9.24% muscle wasting, as determined by the previously calculated coefficient of variation, based on the reproducibility of the measure in preliminary studies [17].

### Plasma microRNA extraction and quantification

In order to provide normalisation for the RNA extraction process we spiked in an exogenous control (*Caenorhabditis elegans* microRNA, C-39). Quantification of microRNAs in plasma and normalisation to a spiked in control has been previously described [21]. RNA was extracted with TRIzol® Reagent (Invitrogen) and miRNAeasy RNA and small RNA extraction kits (Qiagen). MicroRNAs were quantified using NCODE™ VILO™ miRNA cDNA synthesis and sybr-green qPCR kits (Invitrogen). Full details of these methods, including primers used, have been previously published by our group [12]. Samples were excluded if the PCR melt curves suggested multiple products or duplicates were inconsistent. Excluded samples were equally distributed between both groups (non muscle-wasting group 48/380 and in the muscle-wasting group 57/460, *P* = 0.91,chi-squared test).

### Data and statistical analysis

MicroRNA data were non-parametrically distributed; as such the data are presented as median with IQR. Repeated measures Friedman’s test with Dunn’s correction for multiple comparisons (all time points to the pre-operative baseline) was used for within-group analysis. The Kolmogorov-Smirnov (KS) test was used for the between-group analysis of miR-181a fold-change, as the variance was different between the groups. Statistical analysis and figure construction was carried out using Graphpad PRISM 6 (GraphPad Software, San Diego, CA, USA). Receiver operating characteristic (ROC) curve analysis and calculation of specificity and sensitivity were carried out using Graphpad PRISM 6. Positive predictive value and negative predictive value were calculated using www.medcalc.org.

## Results

### Patients

The demographic data and clinical characteristics of this patient cohort have been previously published [17]. Of the 42 patients included in the data analysis 55% (n = 23) developed muscle wasting. Those who developed muscle wasting and those who did not were well-matched in terms of age, sex, body mass index, co-morbidities and surgical risk (EuroSCORE) at baseline (Table 1), indicating that these factors cannot be used to predict skeletal muscle loss. Clinical data for the two groups of patients are shown in Table 1; the median length of ICU stay was 1 day for non muscle-wasting patients and 2 days for those with muscle wasting (*P* = 0.62, Mann-Whitney test). Further clinical data including indication for cardiac surgery can be found in the original study and its online supplement [17].

**Table 1:** **Demographic data, co-morbidities and critical care data for non muscle-wasting (n = 19) and muscle-wasting patients (n = 23)**

	**Non muscle-wasting patients(n = 19)**	**Muscle-wasting patients (n = 23)**	***P*** **-value (** ***t*** **-test, Mann-Whitney** ^**#**^ , chi-squared*)
**Demographic data**			
Age, years	65.7 ± 17.2	62.0 ± 16.2	0.47
Sex, male/female	9/10	12/11	0.76*
Body mass index, kg/m2^a^	26.6 ± 4.6	24.7 ± 4.1	0.18
EuroSCORE	5.3 ± 1.8	5.5 ± 2.6	0.76
Pre-operative creatinine, umol/L^a^	81.3 ± 23.6	77.3 ± 17.1	0.52
Pre-operative creatinine clearance, ml/minute^a^	84.7 ± 36.1	86.8 ± 34.7	0.66
**Pre-operative co-morbidities, n**			
Ischaemic heart disease	10	8	0.24*
Structural heart disease	9	11	0.97*
Dysrhythmia	2	3	0.80*
Systemic hypertension	7	5	0.94*
Pulmonary hypertension	1	2	0.66*
Hypercholesterolaemia	7	10	0.66*
Congenital cardiac disease	3	6	0.42*
Diabetes	3	1	0.21*
Obstructive lung disease	3	2	0.45*
**Outcome data**			
C-reactive protein day 2	165 ± 60	189 ± 68	0.23
ICU length of stay, days, median (IQR)	1 (1 to 2.5)	2 (1 to 3)	0.62^#^
Hospital length of stay, days, median (IQR)	8 (7 to 11)	9 (8 to 10.5)	0.44^#^

Data presented as mean ± SD unless otherwise stated. ^a^Weight unavailable for two patients in the muscle-wasting group, therefore, n = 21. Data adapted from previously published study [17]. p values calcultated by t-test, # - Man-Whitney or * - Chi-squared as appropriate.

### MicroRNAs

**Figure 1:**
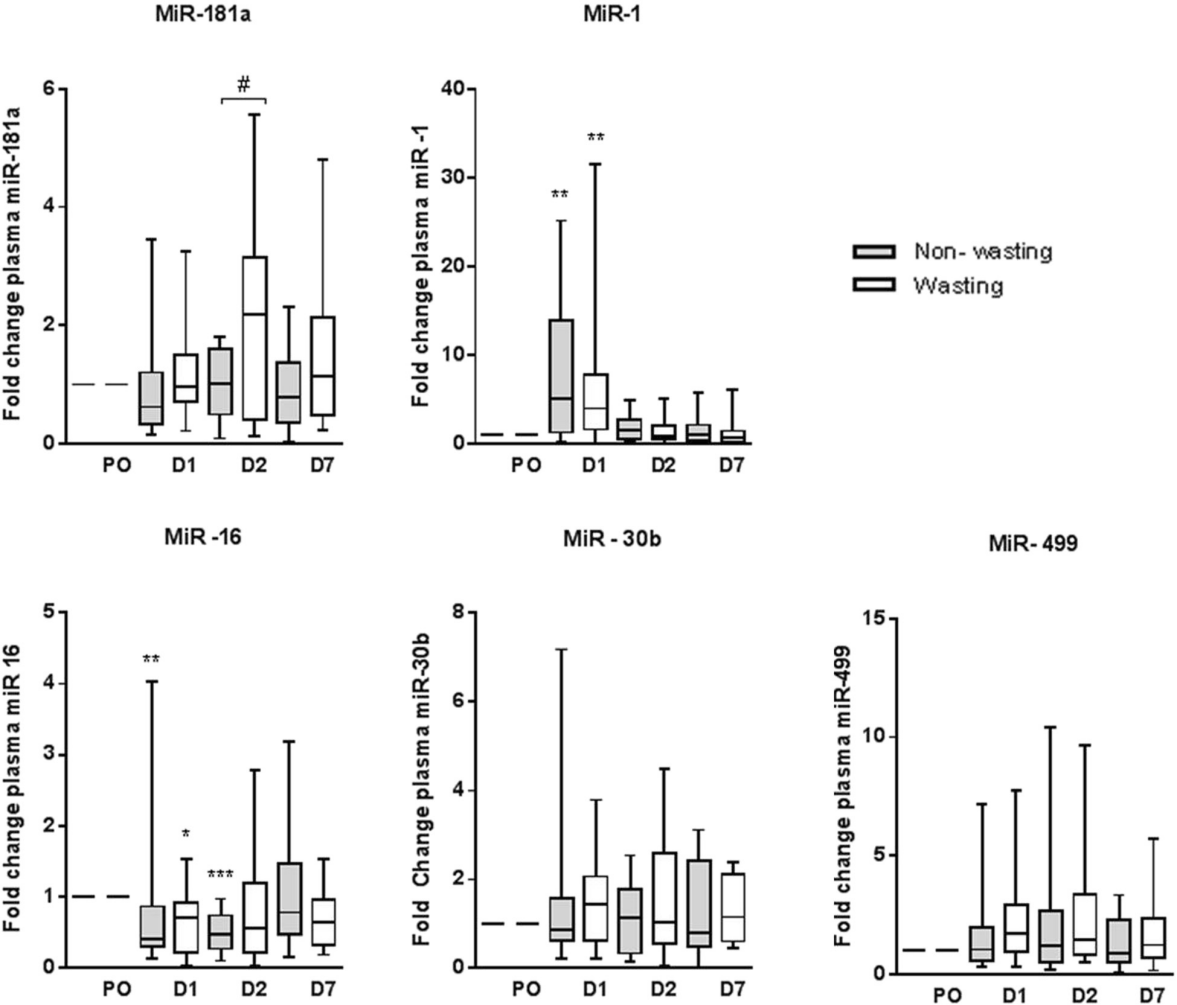
**Relative plasma microRNA concentration in patients with and without muscle wasting in adult critical care.** Relative plasma microRNA concentration in non muscle-wasting (n = 19) and muscle-wasting patients (those with >9.24% muscle loss; n = 23) pre-operatively (PO), on day 1 (D1), day 2 (D2) and on day 7 (D7). Data presented as box and whisker plots with median, interquartile ranges and 5 to 95% percentiles. *P <0.05, **P <0.01, ***P <0.001, repeated measures Freidman’s test with Dunn’s correction for comparison with pre-operative baseline. ^#^ *P* = 0.03 for comparison between groups with the Kolmogorov-Smirnov test.

miR-133 and -206 were not detectable. miR-16 was quantified as a control microRNA [21]. No statistical difference between muscle-wasting and non muscle-wasting patients was found in plasma concentration of these microRNAs at baseline, or in the absolute values at any other time point. Repeated measurements were therefore expressed and analysed relative to the patient’s own baseline (fold-change). miR-30b and -499 remained unchanged both between and within groups over time (Figure 1).

### miR-181a

Median fold-change in plasma miR-181a at D2 was 2.15 (IQR 0.40 to 3.15) in those who developed muscle wasting compared to 1.02 (IQR 0.50 to 1.60) in those who did not (*P* = 0.03) (Figure 1). ROC curve analysis for the increase in D2 miR-181a gave an area under the curve of 0.6. A rise in miR-181a at D2 greater than 1.7 times baseline gave a specificity of 91% and sensitivity of 56% for acute muscle wasting. This cut off resulted in a positive predictive value of 91% and a negative predictive value of 56%^a^.

### miR-1

miR-1 was significantly elevated at D1 compared to baseline in both groups (median 4.03 times higher (IQR 1.65 to 7.84) in patients with muscle wasting and 5.14 (IQR 1.31to 14.00 in patients with no muscle wasting); *P* <0.01 for both). The fold-change increase in plasma miR-1 was not different between the two groups. miR-1 returned to baseline by D2 in both groups (Figure 1).

### miR-16

miR-16 was significantly reduced at D1 compared to baseline in both groups (median 0.71 of baseline (IQR 0.21 to 0.92) in patients with muscle wasting, *P* <0.01, and median 0.41 of baseline (IQR 0.31 to 0.87) in patients with no muscle wasting, *P* <0.05). The fold-change reduction was not different between the groups and returned to baseline in both (Figure 1).

## Discussion

We provide preliminary data that show that following elective high-risk cardiothoracic surgery, both patients with and without subsequent muscle wasting exhibited changes in the circulating microRNA profile. Despite the low area under the ROC curve (0.6), we found that a 1.7-fold increase in miR-181a was 91% specific for a subsequent reduction in RF_CSA_, however, its value in excluding a subsequent reduction in RF_CSA_ was less striking. These data are preliminary and as with all biomarker studies should be repeated in a validation cohort, and with more frequent time point measures to define the window after surgery (or admission) with the greatest clinical utility.

### Critique of the method

This is a relatively small study with a limited population of patients. In evaluating new tests it is important to avoid scenarios where there is a high pre-test probability of the condition one is testing for (in this case quadriceps loss assessed by RF_CSA_) to provide a fair evaluation of the test. Our disease model included patients with brief ICU stays and an uncomplicated post-operative course. Nevertheless, we achieved an approximately 50:50 split between those who developed a reduction in RF_CSA_ at the seventh post-operative day and those who did not, suggesting that the model is suitable for evaluating the test. While this model of acute muscle wasting following cardiothoracic surgery allowed us to control for many of the other factors that occur in the critically ill, and thus, investigate a disease marker that is specific to muscle wasting, it remains to be seen whether our results are generalisable to a wider ICU population (including both a greater sample size and wider variation in ICU disease). It may be that these changes are only seen in this limited cohort of patients. It is also important to note that the muscle wasting described here was mild, no patients developed debilitating muscle weakness and none reached the diagnostic criteria for true ICU-acquired weakness as set out by Stevens *et al*. [22]. A measure of muscle strength and the relationship between this and muscle wasting and changes in microRNAs would potentially add to the study, however, we found that the Medical Research Council (MRC) strength score was not discriminatory enough (all but four patients scoring 60/60 at 1 week) and handheld dynamometry was limited by the pain of sternal wounds following surgery; thus, a more detailed strength evaluation would require use of a non-volitional technique such as magnetic nerve stimulation [23]. It is possible that in a sicker group of patients a clearer, stronger or different signal in change in circulating microRNA profile might have been seen.

miR-16 was measured in this study as a control microRNA. It is widely expressed and in plasma has previously been used to demonstrate that findings in more specific microRNAs do not result from general changes in microRNA levels [12,21]. However, miR-16 has recently been shown to be elevated in the plasma of septic patients and is increasingly implicated in disease processes [24]. Our data show a reduction in miR-16 in both groups of patients. These patients were not septic, but all patients did experience an inflammatory insult as evidenced by a significant rise in CRP in both groups peaking at day 2 (Table 1). The differences between inflammation secondary to infection (sepsis) and inflammation without infection, are increasingly being recognised [24] and our data support this. Our data also suggest that miR-16 levels in plasma do change significantly, implying that it is not appropriate as reference microRNA in these circumstances. However, not all microRNAs measured changed overtime and therefore we feel that the changes observed represent real rather than methodological differences. Both groups showed an acute increase in miR-1 at D1 recovering to baseline on D2 after surgery. This may be accounted for by miR-1 being highly expressed in cardiac as well as in skeletal muscle, and all patients underwent cardiothoracic surgery [9]. Plasma miR-1 has been found to be elevated acutely following myocardial damage in acute coronary syndromes in several different clinical studies [9] and to be predictive of outcome [25].

Last, although we chose to examine these microRNAs because of their roles in muscle homeostasis, we do not know, in these patients, how the changes in plasma level of these microRNAs reflect the relative expression or function of these microRNAs in the muscle, and caution is therefore required in drawing mechanistic conclusions. MicroRNAs circulate in the blood in exosomes or bound to protein and high-density lipoproteins. The regulation, specificity and secretion of microRNA into the circulation is not fully understood [9]. In COPD, specific microRNAs change in opposite directions in muscle and plasma, for example, miR-1 was reduced in the muscle of patients with COPD and reflected muscle wasting, whereas it was increased in the plasma of the same patients [12,13], consistent with our observation that miR181a was reduced in the muscle of patients with established ICU-acquired weakness [16]. It is also important to note that as the field of microRNA research increases there may be other, as yet unknown, microRNA that would be better biomarkers than the ones targeted in this work. Furthermore, there is significant variability between individuals and the standard errors are large. The reasons for this are unclear; however, these variations are in line with those seen in the literature, where similar fold-change differences in relatively small numbers of participants are also described [10]. Also, it may be that as our understanding of microRNA regulation in the circulation improves, normalisation of these values, for example to body mass or circulating lipid levels, may become relevant and may allow a normal range be described. However the rise in miR-181a seen here suggests potential directions for future research especially given its established roles in inflammation and muscle metabolism [15,26].

### Clinical perspective

Our data suggest that a rise in miR-181a early in ICU admission may have a high specificity for muscle wasting at 1 week. The high positive predictive value of this test (91%) allows it to be used to identify at-risk patients. However, due to the relatively low sensitivity (56%) not all patients at risk of muscle wasting will be identified, therefore this test could not be used to exclude a diagnosis of acute muscle wasting. Several possible reasons suggest themselves for the poorer sensitivity than specificity of this marker. First, mir-181a may indeed have a mechanistic role; as it is acknowledged that ICU-acquired weakness is multi-factorial then if mir-181a were mechanistic, it would only capture cases in which this mechanism was the major underlying factor. Second, while muscle wasting is long-lasting, both in the ICU and in other clinical scenarios, the miR-181a rise was not. Thus, it is conceptually possible that patients who had weakness at D7 but only a small fold-change in miR-181a at D2 would have had a greater rise had it been measured, for example, at D4. Last, as circulating, microRNA levels depend on export of the microRNA from cells, other factors increasing cell permeability to this miRNA could contribute to the presence of miR-181a in the plasma.

Currently there is no effective therapy for muscle atrophy beyond ensuring adequate nutrition and facilitating early exercise and mobilisation. However, there is increasing interest in preventing muscle loss by the application of external neuromuscular electrical stimulation. Similarly new generation anabolic agents are presently being evaluated in animals, for example, those targeting the myostatin signalling pathway [27,28]. In developing translational studies, which bring novel medicines to patients, it will be useful to stratify patients by the likelihood of developing subsequent muscle loss and circulating miR-181a could be useful for this purpose by reducing the size of a proof-of-concept study.

The biological role of miR-181a is not fully understood. It is expressed in many different tissues and is not muscle specific. However, miR-181a is essential for regeneration and differentiation in muscle after tissue injury [15]. miR-181a is expressed highly in differentiating and regenerating muscle, and by suppression of Homeobox Protein A11 (HOX-A11) it promotes muscle differentiation [15]. Furthermore, in other tissues it has been shown to be involved in the regulation of inflammatory pathways [26,29]. Both of these pathways may be relevant in the response of muscle to critical illness [30]. However, miR-181b has been found to be reduced in blood obtained from ICU patients with sepsis [31]. Sepsis is certainly a major aetiological factor in the development of critical illness-related acute muscle wasting [30] but our patients were not septic. Furthermore, although miR-181a and miR-181b are from the same family of microRNAs, and likely to be co-transcribed, potential differences in their function are not fully understood [15]. miR-181a is the major member of the miR-181 family that has been shown to be functional in human muscle [15].

## Conclusion

In conclusion we have evaluated the potential role of selected microRNAs for the prediction of subsequent muscle loss in patients undergoing elective high-risk cardiothoracic surgery. We found that an elevation of miR-181a may represent a specific marker of quadriceps wasting, which deserves further prospective evaluation in larger unselected populations of critically ill patients.

## Key messages

- Acute muscle wasting in the critically ill is difficult to accurately predict and as such the search for a biomarker of acute muscle wasting in these patients is important.
- We identify miR-181a as a potential specific biomarker of acute muscle wasting in the critically ill that deserves further prospective evaluation.

## Endnote

^a^Of note, in this case the positive predictive value and specificity, and the negative predictive value and sensitivity are the same numerically because the number of true positives and true negatives are numerically the same.

## Abbreviations

COPD: chronic obstructive pulmonary disease
KS: Kolmogorov-Smirnov
NIHR: National Institute for Health Research
MRC: Medical Research Council
NHS: National Health Servcie
ROC: receiver operating characteristic
US RF_csa_: ultrasound cross-sectional area of the rectus femoris muscle
